# Closed Reduction of an Acute Volar Dislocation of the Distal Radio-Ulnar Joint by a Modified Technique

**DOI:** 10.1155/2018/4289406

**Published:** 2018-08-02

**Authors:** Samuel Larrivée, Graeme Matthewson, Laurie Barron

**Affiliations:** ^1^Department of Surgery, Section of Orthopedics, Faculty of Medicine, University of Manitoba, Winnipeg, MB, Canada; ^2^Pan Am Clinic, Winnipeg, MB, Canada

## Abstract

There is scarce literature describing treatment of volar dislocation of the distal radio-ulnar joint (DRUJ). Irreducible dislocation is usually treated surgically. We present the case of a 37-year-old male with acute right wrist pain and loss of pronation. A diagnosis of volar DRUJ dislocation was made. Reduction using conventional technique was unsuccessful. A second attempt was successful by applying pressure over the interosseous membrane of the forearm and manipulating the ulnar head. At three weeks, the patient had minimal pain, a stable DRUJ, and near complete range of motion. This modified technique for reduction of a locked anterior DRUJ dislocation can be used to avoid an unnecessary surgical intervention.

## 1. Introduction

Volar dislocation of the distal radio-ulnar joint (DRUJ) is a rare occurrence in the literature, with only 35 cases reported in the literature. Multiple methods of reduction and fixation have been described, ranging from closed reduction to open reduction and internal fixation with suture anchors [1, 2]. There are several anatomic blocks that can be encountered when attempting a reduction including impaction of the ulnar head, spasm of the pronator quadratus, and interposition of the torn triangular fibrocartilage complex (TFCC) [3–5]. Out of 35 cases, there have been only five cases of ulnar head impaction reported, each necessitating an open reduction [1, 3, 6–8]. Here, we describe the case of a volar DRUJ dislocation with an ulnar head impaction fracture reduced by closed reduction using a modified technique.

## 2. Case Presentation

### 2.1. Presentation and Clinical Findings

**Figure 1:**
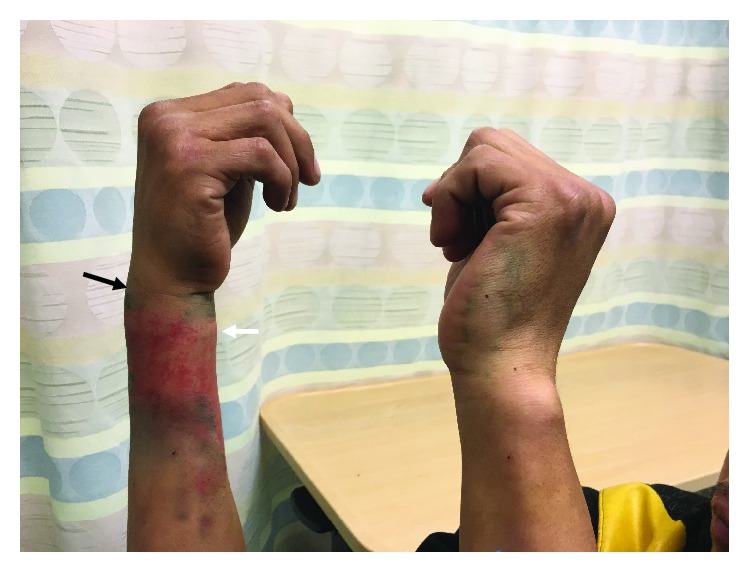
A clinical photograph of the patient's deformity. Notice the loss of the dorsal bulge of the ulnar head (black arrow) and the fullness of the volar aspect of the right wrist (white arrow). Of note, the patient's multiple tattoos were digitally removed to reduce the risk of identification.

**Figure 2:**
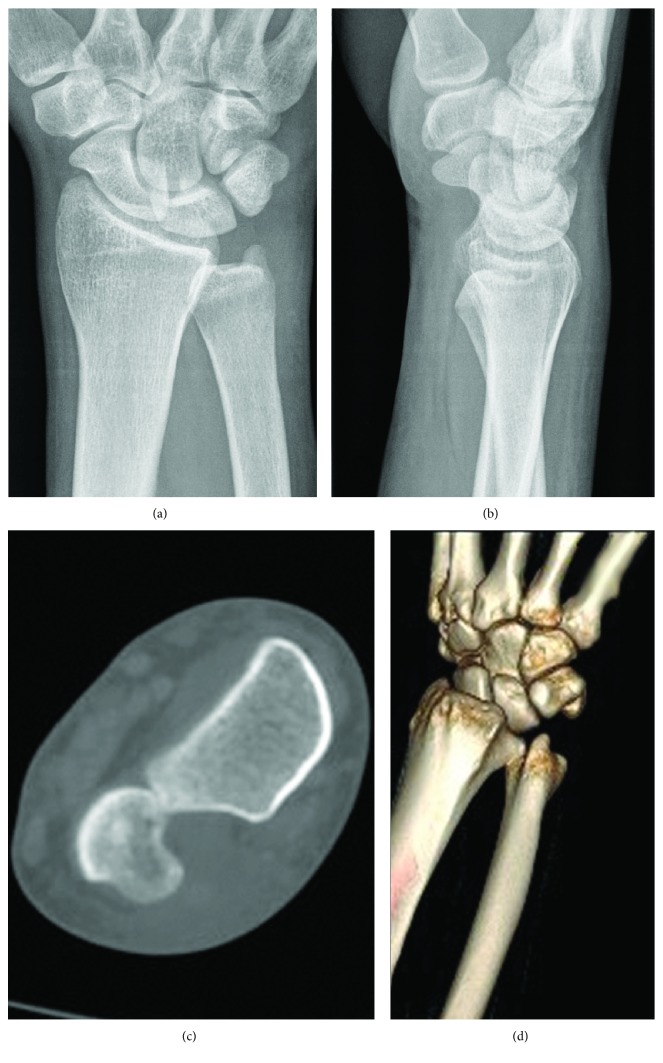
AP (a) and lateral (b) radiographs of the injured wrist. Notice overlap between the radius and ulna on the AP view and slight anterior position of the ulna in relation to the carpal bones on the lateral view. Select coronal cut (c) and 3D reconstruction (d) of the forearm clearly show that the ulnar head is dislocated outside of the sigmoid notch and impacted on the radius.

A 37-year-old right-handed male presented to the emergency department with right wrist pain and decreased range of motion of the forearm following a friendly grappling match. On history, he was mildly intoxicated by alcohol at the time of the injury. His friend had performed an arm-bar holding the patient's right arm between his legs while pulling on the forearm with his hands. The patient tried to escape the maneuver by forcefully pulling and pronating his forearm. He immediately felt pain in his right forearm and was unable to use it afterwards. His past medical history was significant for alcohol, tobacco, and cocaine use. He was not known to be suffering any other medical conditions and had never suffered any injury to his right wrist or forearm in the past. On physical examination, the forearm was locked in supination, with no passive or active pronation elicited. There was loss of the dorsal ulnar prominence, and a palpable and tender solid mass was felt on the volar aspect of the wrist, which was presumed to be a volarly dislocated ulnar head (Figure 1). The skin was intact, and the neurovascular status of the hand was normal. Radiographic examination of bilateral forearms confirmed our suspicions, displaying overlap between the radius and ulna on the anteroposterior view and volar displacement of the ulnar head relative to the distal radius on the lateral view. A CT scan was performed, completing the clinical picture by revealing impaction of the ulnar head on the distal radius (Figure 2).

### 2.2. Treatment

**Figure 3:**
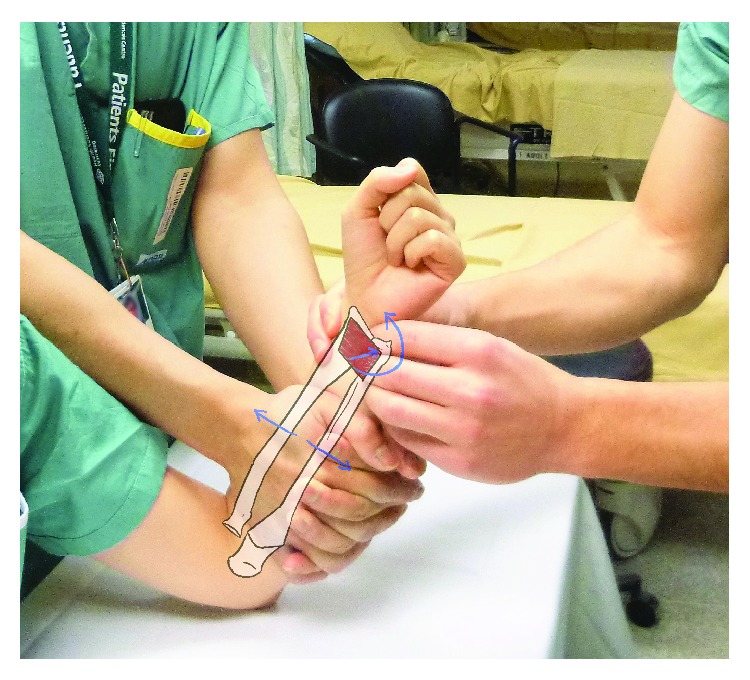
Reenactment of the reduction technique. An assistant applies pressure on the interosseous membrane (IOM) of the forearm, while the physician pushes posteriorly on the ulnar head and pronates the forearm.

**Figure 4:**
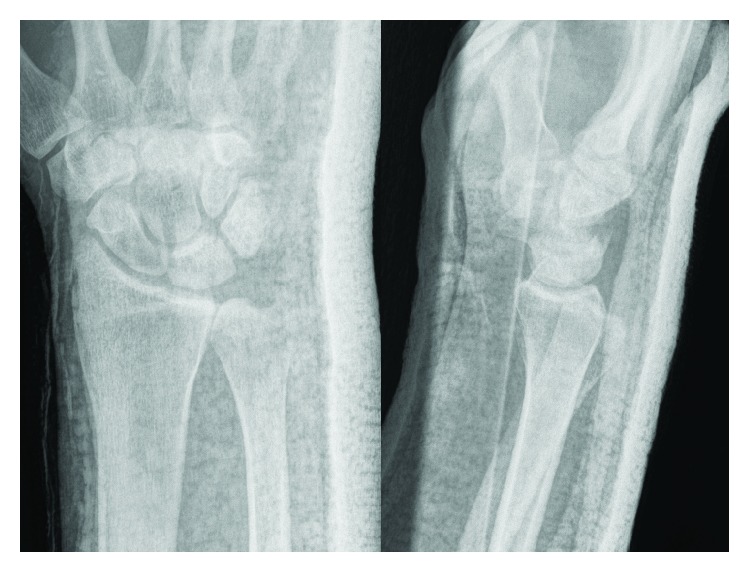
Postreduction radiographs of the right wrist. There is no more overlap between the radius and the ulna on the AP view, and the ulnar head is now slightly dorsal to the radius on the lateral view.

After obtaining informed consent, closed reduction was planned under procedural sedation. The reduction was first attempted by pronating the forearm while applying a posteriorly directed force to the ulnar head. After an unsuccessful first attempt, a second attempt was performed with an assistant applying pressure on the interosseous membrane (IOM) of the forearm using the palm of both of his hands, in an effort to free the impacted ulnar head from the distal radius (Figure 3). With the ulnar head now freed from the radius, the second attempt at manipulation was rewarded by an audible click and a return of the wrist's normal position and motion. On postreduction stability testing, the DRUJ was felt to be unstable at 45° of supination. This prompted the clinician to immobilize the patient in neutral rotation and 90° of flexion using an above-elbow back slab. Postreduction radiographs confirmed the success of the reduction maneuver (Figure 4).

### 2.3. Outcome

At the three-week clinical follow-up, the splint was removed and physical examination was repeated. The patient showed full range of motion of the wrist and elbow in flexion and extension. Compared to the contralateral forearm, there was a 10° lack of pronation and 25° lack of supination. Pain and tenderness were minimal, and no instability could be elicited. Diagnostic imaging confirmed that the reduction was maintained. The patient was discharged from the clinic with instructions for range of motion exercises and avoidance of loading activities for an additional three weeks. No additional follow-up visits were planned.

## 3. Discussion

Volar dislocation of the DRUJ is infrequently encountered in practice, with approximately 35 cases reported. Thus, the diagnosis can be initially missed in the emergency department, leading to chronic disability [9]. The primary stabiliser of the DRUJ is the TFCC, which encompasses the dorsal and volar radio-ulnar ligaments, the central articular disc, the meniscus homolog, the extensor carpi ulnaris (ECU) subsheath, and the ulnocarpal ligaments. Secondary stabilisers include static stabilisers such as the bony sigmoid notch, the joint capsule, and the IOM, and dynamic stabilisers such as the ECU and pronator quadratus [10]. The IOM seems particularly essential to prevent volar DRUJ dislocation. Its distal portion is taut through pronation and supination, while its middle and proximal portions are taut in neutral position and maximally relaxed in supination [11]. In a biomechanical analysis of DRUJ instability, Watanabe et al. showed that both a TFCC tear and a rupture of the proximal IOM were necessary to produce a dorsal dislocation of the radius relative to the ulna (more commonly referred to as a volar DRUJ injury) [12].

The usual causal mechanisms of a volar DRUJ dislocation include a fall on an outstretched hand [3, 13–15], direct blow on the wrist [7, 8, 16], or hypersupination of the forearm [2, 4, 9, 17–22]. This injury can be associated with more complex patterns, such as a radius fracture in the Galeazzi injury or a complete disruption of the IOM and radial head fracture as seen in the Essex-Lopresti lesion [23]. On physical examination, the wrist may appear narrowed, with an unusual fullness felt on the anterior aspect of the wrist. Most noticeable findings include a loss of the dorsal ulnar prominence and a wrist that is locked in supination [24]. True anteroposterior (AP) and lateral radiographic views of the forearm should point to the accurate diagnosis, showing slight overlap between the radius and ulna on the AP view and anterior position of the ulna in regard to the carpal bones on the lateral view. As these signs might be easily missed, a CT scan of the forearm and wrist should be ordered if there is any suspicion [15]. For most simple cases, anterior DRUJ dislocation can be treated successfully with closed reduction, followed by an above-elbow cast for a duration of three to six weeks [2, 14, 15, 18, 22, 25, 26]. Reduction, however, can be made more difficult by different blocks to reduction, such as impaction of the ulnar head [1, 3, 6–8], spasm of the pronator quadratus [5, 22], and interposition of the torn TFCC [4]. In cases of a failed reduction, additional imaging (CT or MRI) could help delineate damage to ligamentous tissue and assist in developing an appropriate treatment plan, which could include open reduction, repair of the TFCC, or reconstruction of the IOM [2, 10, 23]. In all previously described cases of ulnar head impaction, an open reduction technique was necessary [1, 3, 6–8]. The idea of a distraction maneuver is not completely new. It was reported that Boyer used his fingers, placed between the radius and ulna at the volar and dorsal aspects of the wrist, distracting the bones from each other to assist in the reduction [24]. A similar technique was employed successfully by Bouri et al. in a case involving spasm of the pronator quadratus; however, they failed to describe the technique used to distract the ulnar head from the radius [22]. Applying pressure over the IOM to achieve distraction is a technique that is easy to perform with an assistant and does not require significant strength. This technique relies on there being a tear to the distal aspect of the IOM and relaxation of the middle and proximal portions in supination. It has proven to be effective in our case in which the ulnar head was impacted on the radius and could be potentially useful to overcome spasm of the pronator quadratus. More widespread use of this technique could help prevent the unnecessary risks associated with a surgical intervention.

## 4. Conclusion

Applying pressure over the IOM in an effort to distract the impacted ulna from the radius proved successful in reducing this rare case of anterior DRUJ dislocation. This modification of the standard reduction technique has, to our knowledge, not been described in the literature to date. The technique can be easily applied with the help of an assistant and avoids the risks associated with open reduction.
